# Molecular phylogeny and morphology reveal two new species, *Ophiocordyceps ramosa* sp. nov. (Ophiocordycipitaceae) and *Leptobacillium hepiali* sp. nov. (Cordycipitaceae), in Hypocreales from China

**DOI:** 10.3897/mycokeys.127.173361

**Published:** 2026-01-23

**Authors:** Juan Shen, Wei Gou, Hui-Ling Tian, Gang-Xiang Fei, Jing-Qiang Guan, Chun-Yan Long, Xiao Zou

**Affiliations:** 1 Institute of Fungus Resources, Key laboratory of Plant Resource Conservation and Germplasm Innovation in Mountainous Region (Ministry of Education), Institute of Agro-Bioengineering, College of Life Sciences, Guizhou University, Guiyang, Guizhou, 550025, China Institute of Agro-Bioengineering, College of Life Sciences, Guizhou University Guiyang China; 2 Guizhou Key Laboratory of Agricultural Microbiology, College of life Sciences, Guizhou University, Guiyang, Guizhou, 550025, China College of life Sciences, Guizhou University Guiyang China; 3 Guizhou Dashahe Nature Reserve Administration Bureau, Zunyi, 563100, Guizhou, China Guizhou Dashahe Nature Reserve Administration Bureau Zunyi China

**Keywords:** *
Leptobacillium
*, new species, *
Ophiocordyceps
*, phylogenetic analysis, taxonomy

## Abstract

*Ophiocordyceps* holds significant value in controlling arthropod populations, maintaining ecosystem balance, and developing bioactive substances. During the natural infection of hosts, *Ophiocordyceps* fungi, along with other fungal species, form a micro-ecological complex, where these microorganisms also exhibit ecological functions and biological value. In this study, two new species were introduced, from *Ophiocordyceps* and *Leptobacillium*, based on morphological observation and multi-gene (ITS, nrSSU, nrLSU, *tef-1α*, *rpb1*, and *rpb2*) phylogenetic analysis. The phylogenetic analysis revealed that *Ophiocordyceps ramosa***sp. nov**. is closely related to *Hirsutella satumaensis* and *H. nodulosa*, and *Leptobacillium hepiali***sp. nov**. is closely related to *L. latisporum*. *O. ramosa***sp. nov**. is characterised by multi-branched or partially forked, lignified and light brown stromata, phialides with a swollen base and tapering neck, and spherical or sub-spherical conidia, distinguishing it from closely related species. *L. hepiali***sp. nov**. is characterized by solitary, unbranched phialides, clearly differentiating it from its relatives, which have 2–3 branched phialides. The distinctiveness of these two new species was strongly supported by both molecular phylogeny and morphology.

## Introduction

The genus *Ophiocordyceps* is one of the most important and diverse genera of entomopathogenic fungi, with significant scientific and practical value. *Ophiocordyceps* was established by Petch (1931) to classify the species exhibiting non-disarticulating ascospores and clavate asci with thickened apices, including *O. blattae*, *O. unilateralis*, *O. rhizoidea* and *O. peltate* with *O. blattae* designated as the type species (Petch 1931). Subsequently, researchers incorporated it into a subgenus of *Cordyceps* (Kobayasi 1941, 1982; Mains 1958). *Ophiocordyceps* was reclassified as a distinct genus in the family Ophiocordycipitaceae, based on morphological and phylogenetic analysis (Sung et al. 2007a). The International Code of Nomenclature for Algae, Fungi and Plants was promulgated in 2012, aligning with the “one fungus, one name” principle. Quandt et al. (2014) proposed to abolish asexual generic names (*Cordycepioideus*, *Didymobotryopsis*, *Didymobotrys*, *Hirsutella*, *Hymenostilbe*, *Mahevia*, *Paraisaria*, *Sorosporella*, *Syngliocladium*, *Synnematium*, *Trichosterigma* and *Troglobiomyces*) and to consolidate them under the revised *Ophiocordyceps*. However, names of several *Hirsutella* species remain unamended (Quandt et al. 2014; Spatafora et al. 2015). *Ophiocordyceps* was divided into four clades: *O. ravenelii* clade, *O. unilateralis* clade, *O. sobolifera* clade and *O. sphecocephala* clade (Sanjuan et al. 2015). The classification was subsequently revised to establish the current taxonomic framework of *Ophiocordyceps*, comprising the *Hirsutella* clade, *O. sobolifera* clade, *O. sphecocephala* clade and *O. ravenelii* clade (Mains 1958; Sung et al. 2007a; Quandt et al. 2014; Sanjuan et al. 2015; Simmons et al. 2015; Wang et al. 2018).

Ophiocordycipitaceae comprises over 500 species, of which more than 300 belong to the genus *Ophiocordyceps*, making it the largest genus in the family (https://indexfungorum.org/, retrieval on 28 September 2025). *Ophiocordyceps* species exhibit diverse morphological characteristics, hosts and habitats. Most species in *Ophiocordyceps* possess tough, wiry, fibrous or flexible, and darkly pigmented stromata. The perithecia are either superficial or completely immersed in the stroma, and are typically arranged ordinally or sometimes obliquely. Ascospores in this genus are predominantly cylindrical and multiseptate, fragmenting into secondary ascospores or remaining intact upon release (Sung et al. 2007a; Xiao et al. 2023). The conidiation is synnematous. The phialides typically feature a swollen base that tapers abruptly into a slender neck, bearing a single conidium or 2–3 conidia embedded in mucus. The hyaline conidia exhibit a variety of morphologies, ranging from citriform, oblong, subcylindrical, globose, rhombic, or reniform (Quandt et al. 2014; Luangsa-ard et al. 2017). Fungal communities associated with *Ophiocordyceps* species and their microecological environments play crucial roles in population differentiation, environmental adaptation, infection mechanisms, and the secretion of bioactive metabolites (Xia et al. 2015, 2016). *Paecilomyces hepiali*, isolated from *Ophiocordyceps* sinensis, produces bioactive compounds and therapeutic effects similar to *O. sinensis*, while *Clonostachys ochroleuca*, also isolated from *O. sinensis*, has been reported to produce cordycepin (Min et al. 2019; Zhang et al. 2019). The fungal community associated with natural *O. sinensis* plays a critical ecological role in its natural habitat. Studies have shown that members of this community, including *Trichoderma* spp., *Archaeorhizomyces*, *Sebacina*, insect pathogenic fungi and mycorrhizal fungi, exert significant ecological functions, and the synergistic interactions among these fungi contribute to the development of *O. sinensis* (Zhang et al. 2010, 2020a). Furthermore, endophytic fungi representing multiple genera, including *Beauveria*, *Leptobacillium*, *Tolypocladium*, and *Fusarium*, have been isolated from *O. indica*, suggesting that these fungi may influence the development of *O. indica* (Sharma et al. 2023).

Species of the genus *Leptobacillium* have a wide range of sources, having been discovered in various substrates, including plants, soil, air, and mural paintings. *L. coffeanum* was an endophytic fungus in coffee plants in Brazil, and *Leptobacillium* sp. Sl27 was reported to promote the growth of tomato as a plant endophyte. Researchers have also isolated *L. leptobactrum* and *L. chinense* from *Ophiocordyceps* spp. (Zare and Gams 2016; Sun et al. 2019; Leplat et al. 2022; Preedanon et al. 2023; Sharma et al. 2023; Liu-Xu et al. 2025; Lu et al. 2025). Zare and Gams (2016), in the revision of the former *Verticillium* Nees section *Albo-erecta*, established the genus *Leptobacillium*. The name “*Leptobacillium*” refers to its narrow conidia. *L. leptobactrum* comprised two varieties: *L. leptobactrum* var. *calidius* and *L. leptobactrum* var. *leptobactrum* (Zare and Gams 2016). The genus *Leptobacillium* contains 11 species (https://indexfungorum.org/, retrieval on 28 September 2025): *L. cavernicola*, *L. chinense*, *L. coffeanum*, *L. filiforme*, *L. latisporum*, *L. leptobactrum*, *L. longiphialidum*, *L. marksiae*, *L. muralicola*, *L. symbioticum*, and *L. xianyushanense*. In most *Leptobacillium* species, conidiogenous cells are solitary, whereas in *L. latisporum* they often branch, producing two or three phialides (Preedanon et al. 2023; Lu et al. 2025). Species of *Leptobacillium* exhibit two types of conidia forming chains: nearly spherical or elliptical conidia located at the apex of long chains, and narrow cylindrical (rod-shaped) to fusiform conidia occurring within long chains (Zare and Gams 2016; Leplat et al. 2022).

This study identifies and describes two new fungal species from China through morphological and phylogenetic analyses, discussing morphological distinctions between the two new species and their related species. One fungus, *Ophiocordyceps ramosa*, parasitizes Lepidoptera larvae, while the other was identified as *Leptobacillium hepiali*. The objective of this study is to reveal their taxonomic placements based on phylogenetic analysis and morphology.

## Materials and methods

### Specimen collection

*Ophiocordyceps* specimens were collected in China. One specimen was collected from Xifeng County, Tieling City, Liaoning Province (42°42.41'N, 124°26.08'E, 228 m), and another specimen was collected from Guizhou Dashahe National Nature Reserve, Zunyi City, Guizhou Province (29°04.98'N, 107°34.89'E, 1073 m).

### Fungal isolation, culture and morphological observations

Isolation: The surface of the infected insect was disinfected with 75% ethanol for 1–3 minutes, followed by rinsing with sterile water. The internal sclerotia were aseptically transferred onto potato dextrose agar (PDA) with penicillin (70 μg/mL) and streptomycin (50 μg/mL). Isolates were cultured at 20 °C in the dark. The purified isolates were transferred to PDA slant medium and stored at 4 °C for preservation at the Institute of Fungus Resources, Guizhou University (GZUIFR).

Culture and Morphological Observations: The slant cultures were inoculated onto PDA solid medium using a sterile needle and incubated at 20 °C for 15–30 days. Colony morphology was documented, and micro-morphology was observed using an Olympus DP23 microscope.

### DNA extraction, polymerase chain reaction (PCR) amplification, and sequencing

DNA was extracted using the Fungal Genomic DNA Extraction Kit, Centrifuge Column Type (Beijing Solabio Technology Co., Ltd.). Five loci including the partial large subunit rRNA (LSU), internal transcribed spacer including the 5.8S rDNA (ITS), the partial small subunit rRNA (SSU), the translation elongation factor 1-alpha gene (*tef-1α*) and the partial RNA polymerase II largest subunit (*rpb1*) were selected for fungal amplification and sequencing (Wang et al. 2015a; Araújo et al. 2018). The PCR followed protocols described by Wang et al. (2015a) and Araújo et al. (2018), and was used to amplify genetic markers using the following primer pairs: LROR/LR5 for the partial nrLSU (Vilgalys and Sun 1994); ITS1/ITS4 for the ITS region (White et al. 1990); nrSSU-CoF/nrSSU-CoR for the SSU (Wang et al. 2015b); and EF1α-EF/EF1α-ER for the *tef1-α* (Bischoff et al. 2006; Sung et al. 2007b), RPB1-5’F/RPB1-5’R for the *rpb1* (Bischoff et al. 2006; Sung et al. 2007b). PCR products were visualized via 1.0% agarose gel electrophoresis and subsequently sent to Sangon Biotech (Shanghai) Co., Ltd. for purification and analysis.

### Phylogenetic analysis

**Figure 1.**
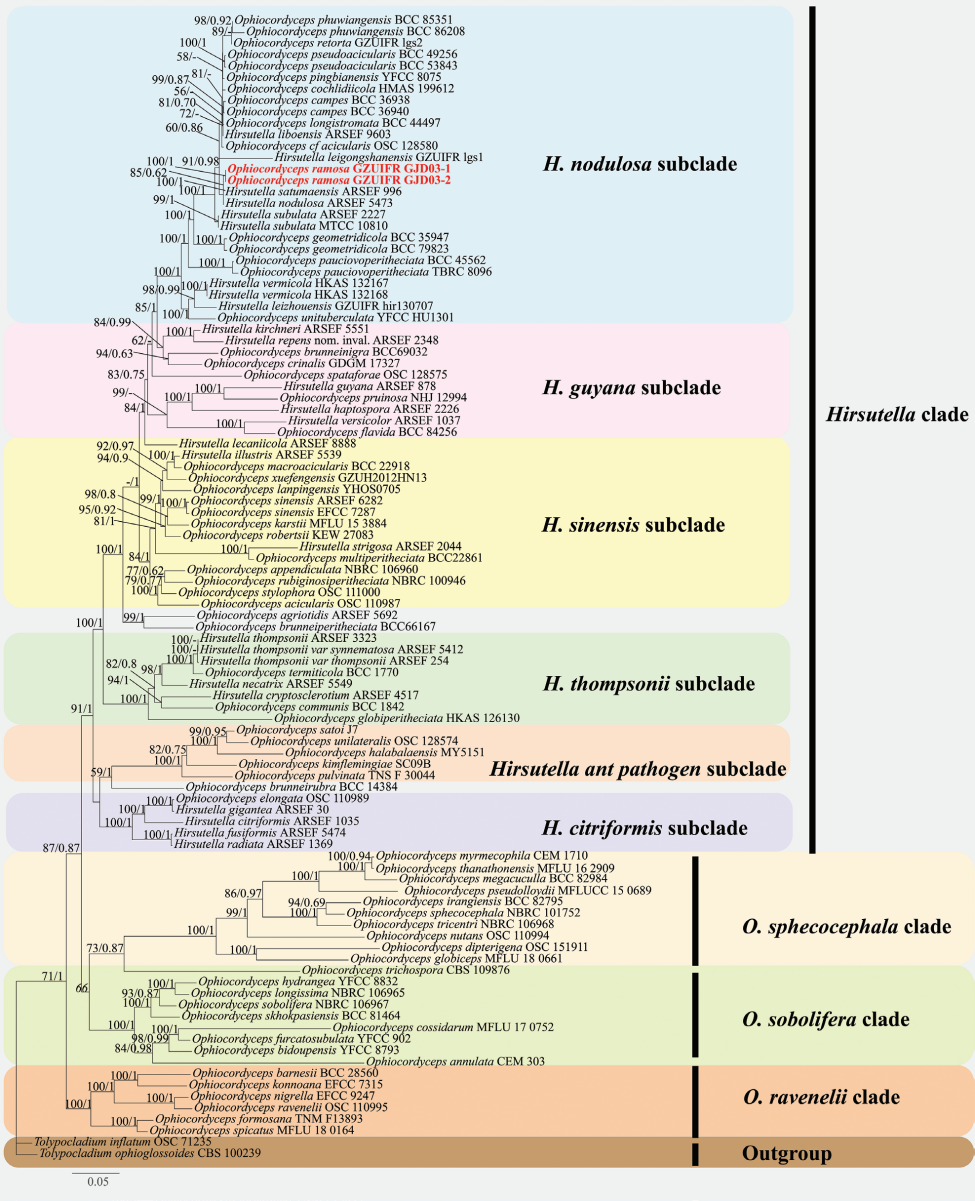
The phylogeny of *Ophiocordyceps* with emphasis on *O. ramosa* and their related species based on six-locus (ITS, nrLSU, nrSSU, *tef-1α*, *rpb1* and *rpb2*) datasets.

**Figure 2.**
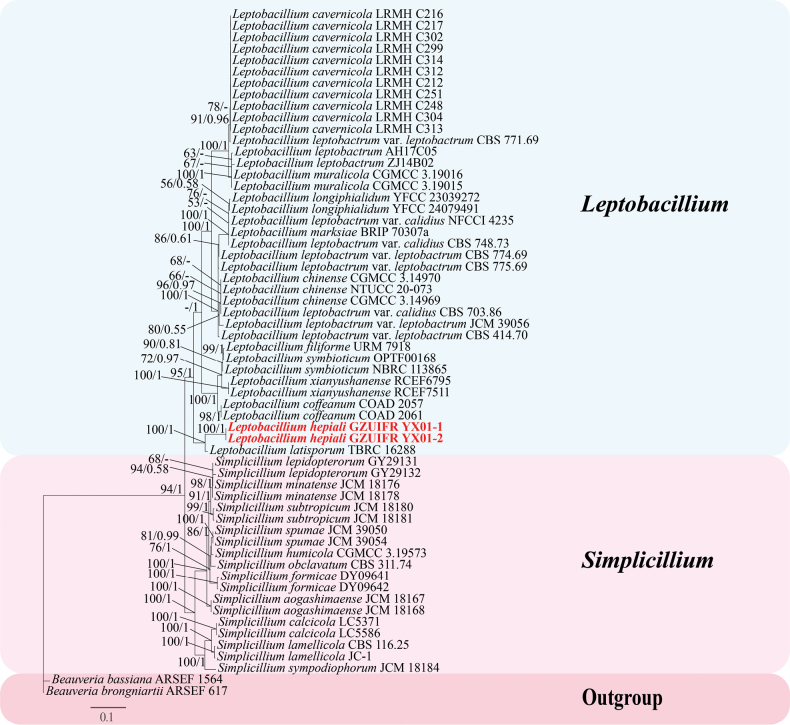
Phylogenetic relationships of *Leptobacillium* and *Simplicillium* based on the combined dataset of ITS, nrLSU, nrSSU, *tef-1α*, *rpb1* and *rpb2*.

The initial sequence chromatograms were examined using Chromas V2.6.5. Sequences were selected for forward and reverse sequence assembly using DNAMAN 9.0.1.116 (LynnonBiosoft, USA), prioritizing those that were accurate and free of bimodal peaks. The newly generated sequences were aligned against other sequences obtained from GenBank using MAFFT v 7 (Katoh et al. 2002), followed by trimming of low-quality regions. PhyloSuite v1.2.2 (Zhang et al. 2020b) was employed for serial and combined analysis of six genes (ITS, nrLSU, nrSSU, *tef-1α*, *rpb1*, and *rpb2*), selecting ModelFinder (Kalyaanamoorthy et al. 2017) as the optimal nucleotide substitution model. The dataset was analyzed using Maximum Likelihood (ML) and Bayesian Inference (BI) methods. Phylogenetic trees were constructed using IQ-TREE (Anisimova et al. 2011; Lam-Tung et al. 2015) under the edge-linked partition model with 5,000 ultrafast bootstrap replicates. Bayesian phylogenetic analysis was performed using MrBayes (Ronquist et al. 2012), with Markov Chain Monte Carlo (MCMC) runs of 10 million generations for Fig. 1 and 2 million generations for Fig. 2. The constructed phylogenetic trees were visualized using iTOL v. 7 (https://itol.embl.de/itol.cgi) in the form of maximum likelihood bootstrap proportions (ML-BS) and Bayesian posterior probabilities (BI-BPP). Final graphical adjustments were made using Adobe Illustrator 2021.

## Results

### Phylogenetic analyses

Phylogenetic analysis of *Ophiocordyceps* was constructed with combined six-locus sequence data representing 100 taxa. Two taxa of the genus *Tolypocladium*, *Tolypocladium ophioglossoides* CBS 100239 and *Tolypocladium inflatum* OSC 71235 were employed as an outgroup (Table 1). The results of the phylogenetic analysis showed that the overall topology was consistent with previous studies, identifying four statistically well-supported clades within *Ophiocordyceps*, namely the *Hirsutella*, *O. sphecocephala*, *O. sobolifera*, and *O. ravenelii* clades (Fig. 1). The *Hirsutella* clade comprised six subclades: *H. citriformis* subclade, *H. thompsonii* subclade, *H. nodulosa* subclade, *H. guyana* subclade, *H. sinensis* subclade, and the *Hirsutella* ant pathogen subclade. Two samples (GZUIFR GJD03-1, GZUIFR GJD03-2), newly described as *Ophiocordyceps ramosa*, were placed into *H. nodulosa* subclade. The specimens of *O. ramosa* formed an independent branch (ML bootstrap = 100%, BI posterior probability = 1) and clustered together with *H. satumaensis* and *H. nodulosa*.

**Table 1.** GenBank accession numbers of the taxa used in the phylogenetic analyses of *Ophiocordyceps*.

Species	Voucher information	GenBank accession no.						Reference
		ITS	nrSSU	nrLSU	*tef-1α*	*rpb1*	*rpb2*	
*Hirsutella citriformis*	ARSEF 1035	KM652153	KM652064	KM652105	KM651989	KM652030	–	Simmons et al. 2015
*Hirsutella cryptosclerotium*	ARSEF 4517	KM652157	KM652066	KM652109	KM651992	KM652032	–	Simmons et al. 2015
*Hirsutella fusiformis*	ARSEF 5474	–	KM652067	KM652110	KM651993	KM652033	–	Simmons et al. 2015
*Hirsutella gigantea*	ARSEF 30	–	–	JX566977	JX566980	KM652034	–	Simmons et al. 2015
*Hirsutella guyana*	ARSEF 878	–	KM652068	KM652111	KM651994	KM652035	–	Simmons et al. 2015
*Hirsutella haptospora*	ARSEF 2226	KM652159	–	–	KM651995	KM652036	–	Simmons et al. 2015
*Hirsutella illustris*	ARSEF 5539	KM652160	KM652069	KM652112	KM651996	KM652037	–	Simmons et al. 2015
*Hirsutella kirchneri*	ARSEF 5551	KM652161	KM652070	KM652113	KM651997	–	–	Simmons et al. 2015
*Hirsutella lecaniicola*	ARSEF 8888	KM652162	KM652071	KM652114	KM651998	KM652038	–	Simmons et al. 2015
*Hirsutella leigongshanensis*	GZUIFR-lgs1	KY415574	–	–	KY415589	KY945359	–	Yu et al. 2017
*Hirsutella leizhouensis*	GZUIFR hir130707	–	–	KY415580	KY415587	KY945358	–	Qu et al. 2017
*Hirsutella liboensis*	ARSEF 9603	KM652163	KM652072	KM652115	–	–	–	Simmons et al. 2015
*Hirsutella necatrix*	ARSEF 5549	KM652164	KM652073	KM652116	KM651999	KM652039	–	Simmons et al. 2015
*Hirsutella nodulosa*	ARSEF 5473	KM652165	KM652074	KM652117	KM652000	KM652040	–	Simmons et al. 2015
*Hirsutella radiata*	ARSEF 1369	–	KM652076	KM652119	KM652002	KM652042	–	Simmons et al. 2015
*Hirsutella repens* nom. inval.	ARSEF 2348	KM652167	KM652077	KM652120	KM652003	–	–	Simmons et al. 2015
*Hirsutella satumaensis*	ARSEF 996	KM652172	KM652082	KM652125	KM652008	KM652047	–	Simmons et al. 2015
*Hirsutella strigosa*	ARSEF 2044	KM652174	–	KM652128	KM652011	–	–	Simmons et al. 2015
*Hirsutella subulata*	ARSEF 2227	KM652176	KM652086	KM652130	KM652013	KM652051	–	Simmons et al. 2015
*Hirsutella subulata*	MTCC 10810	KJ524684	–	KJ524705	–	–	–	Simmons et al. 2015
*Hirsutella thompsonii*	ARSEF 3323	KM652188	KM652096	KM652143	KM652024	KM652059	–	Simmons et al. 2015
*Hirsutella thompsonii* var. *synnematosa*	ARSEF 5412	KM652193	KM652100	KM652148	–	–	–	Simmons et al. 2015
*Hirsutella thompsonii* var. *thompsonii*	ARSEF 254	KM652194	KM652101	KM652149	KM652028	KM652062	–	Simmons et al. 2015
*Hirsutella vermicola*	HKAS 132167	PQ423678	PQ424974	PQ423697	PQ569874	PQ569888	PQ569904	Yang et al. 2025
*Hirsutella vermicola*	HKAS 132168	PQ423679	PQ424975	PQ423698	PQ569875	PQ569889	PQ569905	Yang et al. 2025
*Hirsutella versicolor*	ARSEF 1037	–	KM652102	KM652150	KM652029	KM652063	–	Simmons et al. 2015
*Ophiocordyceps acicularis*	OSC 110987	–	EF468950	EF468805	EF468744	EF468852	–	Sung et al. 2007a
*Ophiocordyceps agriotidis*	ARSEF 5692	JN049819	DQ522540	DQ518754	DQ522322	DQ522368	DQ522418	Kepler et al. 2012
*Ophiocordyceps annulata*	CEM 303	–	KJ878915	KJ878881	KJ878962	KJ878995	–	Quandt et al. 2014
*Ophiocordyceps appendiculata*	NBRC 106960	JN943326	JN941728	JN941413	AB968577	JN992462	AB968539	Ban et al. 2015
*Ophiocordyceps barnesii*	BCC 28560	–	EU408776	–	–	EU408773	EU418599	Luangsa-ard et al. 2010
*Ophiocordyceps bidoupensis*	YFCC 8793	–	OM304638	–	OK556894	OK556898	OK556900	Zou et al. 2022
*Ophiocordyceps brunneinigra*	BCC69032	–	–	MF614654	MF614638	MF614668	MF614681	Luangsa-ard et al. 2018
*Ophiocordyceps brunneiperitheciata*	BCC66167	–	–	MF614659	MF614644	–	MF614684	Luangsa-ard et al. 2018
*Ophiocordyceps brunneirubra*	BCC 14384	MH754736	–	MH753690	GU797121	MK751465	MK751468	Tasanathai et al. 2019
*Ophiocordyceps campes*	BCC36938	MT783955	–	MT118175	MT118167	MT118183	MT118188	Tasanathai et al. 2020
*Ophiocordyceps campes*	BCC36940	MT783954	–	MT118176	MT118168	–	MT118189	Tasanathai et al. 2020
*Ophiocordyceps cf. acicularis*	OSC 128580	–	DQ522543	DQ518757	DQ522326	DQ522371	DQ522423	Spatafora et al. 2007
*Ophiocordyceps cochlidiicola*	HMAS 199612	–	KJ878917	KJ878884	KJ878965	KJ878998	–	Quandt et al. 2014
*Ophiocordyceps communis*	BCC 1842	MH754726	–	MH753680	MK284266	MK214110	MK214096	Tasanathai et al. 2019
*Ophiocordyceps cossidarum*	MFLU 17-0752	–	MF398186	MF398187	MF928403	MF928404	–	Hyde et al. 2017
*Ophiocordyceps crinalis*	GDGM17327	–	KF226253	KF226254	KF226256	KF226255	–	Wang et al. 2014
*Ophiocordyceps dipterigena*	OSC 151911	–	KJ878919	KJ878886	KJ878966	KJ879000	–	Quandt et al. 2014
*Ophiocordyceps elongata*	OSC 110989	–	–	EF468808	EF468748	EF468856	–	Sung et al. 2007a
*Ophiocordyceps flavida*	BCC 84256	–	–	MT512655	MT533482	MT533476	–	Mongkolsamrit et al. 2021
*Ophiocordyceps formosana*	TNM F13893	–	KJ878908	–	KJ878956	KJ878988	KJ878943	Quandt et al. 2014
*Ophiocordyceps furcatosubulata*	YFCC 902	–	MT774214	MT774221	MT774242	MT774228	MT774235	Wang et al. 2021
*Ophiocordyceps geometridicola*	BCC 35947	–	–	MF614647	MF614631	MF614664	MF614678	Luangsa-ard et al. 2018
*Ophiocordyceps geometridicola*	BCC 79823	–	–	MF614648	MF614632	MF614663	MF614679	Luangsa-ard et al. 2018
*Ophiocordyceps globiceps*	MFLU 18-0661	MH725816	MH725812	MH725830	MH727388	–	–	Xiao et al. 2019
*Ophiocordyceps globiperitheciata*	HKAS 126130	OR015963	OR082950	OR015968	OR030532	OR119834	–	Fan et al. 2024
*Ophiocordyceps halabalaensis*	MY5151	GU723763	KM655826	–	GU797110	–	–	Luangsa-ard et al. 2011
*Ophiocordyceps hydrangea*	YFCC 8832	–	OM304636	OM304640	OM831277	OM831280	OM831283	Zou et al. 2022
*Ophiocordyceps irangiensis*	BCC 82795	MH028142	–	–	MH028186	MH028164	MH028174	Khonsanit et al. 2019
*Ophiocordyceps karstii*	MFLU 15-3884	–	KU854952	–	KU854945	KU854943	–	Li et al. 2016
*Ophiocordyceps kimflemingiae*	SC09B	–	KX713631	KX713620	KX713698	KX713724	–	Araújo et al. 2018
*Ophiocordyceps konnoana*	EFCC 7315	–	EF468959	–	EF468753	EF468861	EF468916	Sung et al. 2007a
*Ophiocordyceps lanpingensis*	YHOS0705	–	KC417458	KC417460	KC417462	KC417464	KC456333	Chen et al. 2013
*Ophiocordyceps longissima*	NBRC 106965	AB968406	AB968392	AB968420	AB968584	–	AB968546	Ban et al. 2015
*Ophiocordyceps longistromata*	BCC44497	MT783956	–	MT118178	MT118170	–	MT118191	Tasanathai et al. 2020
*Ophiocordyceps macroacicularis*	BCC22918	–	–	MF614655	MF614639	MF614669	MF614675	Luangsa-ard et al. 2018
*Ophiocordyceps megacuculla*	BCC 82984	–	–	MH028162	MH028192	–	MH028181	Khonsanit et al. 2019
*Ophiocordyceps multiperitheciata*	BCC22861	–	–	MF614656	MF614640	MF614670	MF614683	Luangsa-ard et al. 2018
*Ophiocordyceps myrmecophila*	CEM 1710	–	–	KJ878894	KJ878974	KJ879008	–	Quandt et al. 2014
*Ophiocordyceps nigrella*	EFCC 9247	JN049853	EF468963	EF468818	EF468758	EF468866	EF468920	Sung et al. 2007a
*Ophiocordyceps nutans*	OSC 110994	–	DQ522549	DQ518763	DQ522333	DQ522378	–	Spatafora et al. 2007
*Ophiocordyceps pauciovoperitheciata*	BCC45562	–	–	MF614651	MF614634	MF614666	MF614674	Luangsa-ard et al. 2018
*Ophiocordyceps pauciovoperitheciata*	TBRC 8096	–	–	MF614649	MF614636	MF614665	MF614672	Luangsa-ard et al. 2018
*Ophiocordyceps phuwiangensis*	BCC85351	MT783958	–	–	MT118174	MT118187	MT118195	Tasanathai et al. 2020
*Ophiocordyceps phuwiangensis*	BCC86208	–	–	MT118180	MT118172	MT118185	MT118193	Tasanathai et al. 2020
*Ophiocordyceps pingbianensis*	YFCC 8075	MT273118	–	MT270099	MT270097	MT270098	MT273117	Tasanathai et al. 2020
*Ophiocordyceps pruinosa*	NHJ 12994	–	EU369106	EU369041	EU369024	EU369063	EU369084	Johnson et al. 2009
*Ophiocordyceps pseudoacicularis*	BCC 49256	–	–	MF614645	MF614629	MF614662	MF614676	Luangsa-ard et al. 2018
*Ophiocordyceps pseudoacicularis*	BCC 53843	–	–	MF614646	MF614630	MF614661	MF614677	Luangsa-ard et al. 2018
*Ophiocordyceps pseudolloydii*	MFLUCC 15-0689	MF351725	–	–	MF372758	MF372761	–	Xiao et al. 2017
*Ophiocordyceps pulvinata*	TNS-F 30044	AB721302	GU904208	–	GU904209	GU904210	–	Kepler et al. 2011
***Ophiocordyceps ramosa***	**GZUIFR GJD03-1**	**PQ530297**	**PQ517232**	**PQ517339**	**PQ552719**	**PQ868603**	–	**This study**
***Ophiocordyceps ramosa***	**GZUIFR GJD03-2**	**PQ530298**	**PQ517233**	**PQ517340**	**PQ552720**	**PQ868604**	–	**This study**
*Ophiocordyceps ravenelii*	OSC 110995	–	DQ522550	DQ518764	DQ522334	DQ522379	DQ522430	Spatafora et al. 2007
*Ophiocordyceps retorta*	GZUIFR-lgs2	KY415594	KY415596	–	KY415597	–	–	Qu et al. 2018
*Ophiocordyceps robertsii*	KEW 27083	–	–	EF468826	EF468766	–	–	Sung et al. 2007a
*Ophiocordyceps rubiginosiperitheciata*	NBRC 100946	JN943341	JN941705	JN941436	AB968581	JN992439	AB968543	Schoch et al. 2012
*Ophiocordyceps satoi*	J7	–	KX713653	KX713599	KX713683	KX713711	–	Araújo et al. 2018
*Ophiocordyceps sinensis*	ARSEF 6282	KM652173	KM652083	KM652126	KM652009	KM652048	–	Simmons et al. 2015
*Ophiocordyceps sinensis*	EFCC 7287	JN049854	EF468971	EF468827	EF468767	EF468874	EF468924	Sung et al. 2007a
*Ophiocordyceps skhokpasiensis*	BCC 81464	MK632043	MK632128	MK632103	MK632077	MK632170	MK632159	Khonsanit et al. 2019
*Ophiocordyceps sobolifera*	NBRC 106967	AB968409	AB968395	AB968422	AB968590	–	–	Ban et al. 2015
*Ophiocordyceps spataforae*	OSC 128575	JN049845	EF469126	EF469079	EF469064	EF469093	EF469110	Sung et al. 2007a
*Ophiocordyceps sphecocephala*	NBRC 101752	JN943351	JN941696	JN941445	AB968591	JN992430	AB968552	Schoch et al. 2012
*Ophiocordyceps spicatus*	MFLU 18-0164	MK863254	MK863047	MK863054	MK860192	–	–	Zha et al. 2021
*Ophiocordyceps stylophora*	OSC 111000	JN049828	DQ522552	DQ518766	DQ522337	DQ522382	DQ522433	Spatafora et al. 2007
*Ophiocordyceps termiticola*	BCC 1770	GU723780	–	MH753677	MK284264	MK214107	MK214093	Tasanathai et al. 2019
*Ophiocordyceps thanathonensis*	MFLU 16-2909	MF850376	–	MF850377	MF872613	MF872615	–	Xiao et al. 2017
*Ophiocordyceps tricentri*	NBRC 106968	AB968410	AB968393	AB968423	AB968593	–	AB968554	Ban et al. 2015
*Ophiocordyceps trichospora*	CBS 109876	–	AF543766	AF543790	AF543779	AY489669	DQ522457	Sung et al. 2007a
*Ophiocordyceps unilateralis*	OSC 128574	–	DQ522554	DQ518768	DQ522339	DQ522385	DQ522436	Spatafora et al. 2007
*Ophiocordyceps unituberculata*	YFCC HU1301	–	KY923214	–	KY923216	KY923218	KY923220	Wang et al. 2018
*Ophiocordyceps xuefengensis*	GZUH2012HN13	KC631801	KC631787	–	KC631792	KC631797	–	Wen et al. 2013
*Tolypocladium inflatum*	OSC 71235	JN049844	EF469124	EF469077	EF469061	EF469090	EF469108	Kepler et al. 2012
*Tolypocladium ophioglossoides*	CBS 100239	–	KJ878910	KJ878874	KJ878958	KJ878990	KJ878944	Quandt et al. 2014

The Bayesian Inference (BI) and the Maximum Likelihood (ML) phylogenetic trees were constructed with the 60 taxa based on a six-gene dataset for revealing the phylogenetic relationships of *Leptobacillium* and *Simplicillium*. *Beauveria bassiana* ARSEF 1564 and *Beauveria brongniartii* ARSEF 617 served as an outgroup (Table 2). The newly described *Leptobacillium hepiali*GZUIFR YX01-1 and GZUIFR YX01-2 formed an independent, well-supported clade, closely related to *L. latisporum*, with ML bootstrap and BI posterior probability values of 100% and 1, respectively.

**Table 2.** GenBank accession numbers of the taxa used in the phylogenetic analyses of *Leptobacillium*.

Species	Voucher information	GenBank accession no.						Reference
		ITS	nrSSU	nrLSU	*tef-1α*	*rpb1*	*rpb2*	
*Beauveria bassiana*	ARSEF 1564	HQ880761	–	AF373871	HQ880974	HQ880833	HQ880905	Rehner et al. 2011
*Beauveria brongniartii*	ARSEF 617	HQ880782	AB027335	–	HQ880991	HQ880854	HQ880926	Rehner et al. 2011
*Leptobacillium cavernicola*	LRMH C217	MF788211	OM628844	OM628783	OM654334	OM677783	OM654323	Leplat et al. 2022
*Leptobacillium cavernicola*	LRMH C212	OM622523	OM628842	OM628781	OM654332	OM677781	OM654321	Leplat et al. 2022
*Leptobacillium cavernicola*	LRMH C216	OM622524	OM628843	OM628782	OM654333	OM677782	OM654322	Leplat et al. 2022
*Leptobacillium cavernicola*	LRMH C248	OM622525	OM628845	OM628784	OM654335	OM677784	OM654324	Leplat et al. 2022
*Leptobacillium cavernicola*	LRMH C251	OM622526	OM628846	OM628785	OM654336	OM677785	OM654325	Leplat et al. 2022
*Leptobacillium cavernicola*	LRMH C299	OM622527	OM628847	OM628786	OM654337	OM677786	OM654326	Leplat et al. 2022
*Leptobacillium cavernicola*	LRMH C302	OM622528	OM628848	OM628787	OM654338	OM677787	OM654327	Leplat et al. 2022
*Leptobacillium cavernicola*	LRMH C304	OM622529	OM628849	OM628788	OM654339	OM677788	OM654328	Leplat et al. 2022
*Leptobacillium cavernicola*	LRMH C312	OM622530	OM628850	OM628789	OM654340	OM677789	OM654329	Leplat et al. 2022
*Leptobacillium cavernicola*	LRMH C313	OM622531	OM628851	OM628790	OM654341	OM677790	OM654330	Leplat et al. 2022
*Leptobacillium cavernicola*	LRMH C314	OM622532	OM628852	OM628791	OM654342	OM677791	OM654331	Leplat et al. 2022
*Leptobacillium chinense*	CGMCC 3.14969	JQ410323	–	JQ410321	–	–	–	Gomes et al. 2018
*Leptobacillium chinense*	CGMCC 3.14970	JQ410324	–	JQ410322	–	–	–	Gomes et al. 2018
*Leptobacillium chinense*	NTUCC 20-073	MT974199	–	MT974414	–	–	–	Gomes et al. 2018
*Leptobacillium coffeanum*	COAD 2057	MF066034	–	MF066032	–	–	–	Liu and Cai 2012
*Leptobacillium coffeanum*	COAD 2061	MF066035	–	MF066033	–	–	–	Liu and Cai 2012
*Leptobacillium filiforme*	URM 7918	MH979338	–	MH979399	–	–	–	Crous et al. 2018
***Leptobacillium hepiali***	**GZUIFR YX01-1**	**PV953308**	**PV953312**	**PV953316**	**PV974934**	**PV974932**	–	**This study**
***Leptobacillium hepiali***	**GZUIFR YX01-2**	**PV953309**	**PV953313**	**PV953317**	**PV974935**	**PV974933**	–	**This study**
*Leptobacillium latisporum*	TBRC 16288	OP856540	OP850838	OP856529	–	–	–	Preedanon et al. 2023
*Leptobacillium leptobactrum*	AH17C05	PP384754	–	PP380808	–	–	–	Unpublished
*Leptobacillium leptobactrum*	ZJ14B02	PP385689	–	PP381743	–	–	–	Unpublished
*Leptobacillium leptobactrum* var. *leptobactrum*	CBS 771.69	EF641868	EF641852	KU382224	–	–	–	Zare and Gams 2016
*Leptobacillium leptobactrum* var. *leptobactrum*	JCM 39056	LC496868	LC496903	LC496888	LC496918	–	–	Zare and Gams 2016
*Leptobacillium leptobactrum* var. *leptobactrum*	CBS 774.69	MH859421	–	MH871192	–	–	–	Zare and Gams 2016
*Leptobacillium leptobactrum* var. *leptobactrum*	CBS 775.69	MH859422	–	MH871193	–	–	–	Zare and Gams 2016
*Leptobacillium leptobactrum* var. *leptobactrum*	CBS 414.70	MH859773	EF641846	MH871535	–	–	–	Zare and Gams 2016
*Leptobacillium leptobactrum* var. *calidius*	CBS 703.86	EF641866	EF641850	KU382226	–	–	–	Zare and Gams 2016
*Leptobacillium leptobactrum* var. *calidius*	CBS 748.73	EF641867	EF641851	KU382227	–	–	–	Zare and Gams 2016
*Leptobacillium leptobactrum* var. *calidius*	NFCCI 4235	MG786580	–	MG786581	–	–	–	Zare and Gams 2016
*Leptobacillium longiphialidum*	YFCC 24079491	PQ509281	PQ508805	PQ508807	PQ560996	PQ567239	–	Lu et al. 2025
*Leptobacillium longiphialidum*	YFCC 23039272	PQ509282	PQ508806	PQ508808	PQ560997	PQ567240	–	Lu et al. 2025
*Leptobacillium marksiae*	BRIP 70307a	PQ061114	–	PQ047739	–	–	–	Tan and Bishop Hurley (direct submission)
*Leptobacillium muralicola*	CGMCC 3.19015	MH379985	–	MH379999	–	–	–	Sun et al. 2019
*Leptobacillium muralicola*	CGMCC3.19016	MH379986	–	MH380000	–	–	–	Sun et al. 2019
*Leptobacillium symbioticum*	NBRC 113865	LC485673	–	LC506046	–	–	–	Okane et al. 2020
*Leptobacillium symbioticum*	OPTF00168	LC485675	–	LC506047	–	–	–	Okane et al. 2020
*Leptobacillium xianyushanense*	RCEF6795	PV102572	–	PV102574	PV097798	PV097799	PV097803	Chang et al. 2024
*Leptobacillium xianyushanense*	RCEF7511	PV134472	–	PV134531	–	–	–	Chang et al. 2024
*Simplicillium aogashimaense*	JCM 18167	AB604002	–	LC496874	LC496904	–	–	Nonaka et al. 2013
*Simplicillium aogashimaense*	JCM 18168	AB604004	–	LC496875	–	–	–	Nonaka et al. 2013
*Simplicillium calcicola*	LC5586	KU746706	–	KU746752	KX855252	–	–	Zhang et al. 2017
*Simplicillium calcicola*	LC5371	KU746705	–	KU746751	KX855251	–	–	Zhang et al. 2017
*Simplicillium formicae*	DY09641	OR121054	–	OR121057	OR126571	–	–	Unpublished
*Simplicillium formicae*	DY09642	OR121055	–	OR121056	OR126572	–	–	Unpublished
*Simplicillium humicola*	CGMCC 3.19573	NR_172845	–	MK329041	MK336071	–	–	Unpublished
*Simplicillium lamellicola*	JC-1	MT807906	MT807908	MT807907	–	–	–	Unpublished
*Simplicillium lamellicola*	CBS 116.25	AJ292393	–	–	DQ522356	DQ522404	DQ522462	Nonaka et al. 2013
*Simplicillium lepidopterorum*	GY29132	MN006245	–	–	MN022266	MN022274	–	Chen et al. 2019
*Simplicillium lepidopterorum*	GY29131	MN006246	–	–	MN022265	MN022273	–	Chen et al. 2019
*Simplicillium minatense*	JCM 18178	AB603993	LC496894	LC496879	LC496909	–	–	Nonaka et al. 2013
*Simplicillium minatense*	JCM 18176	AB603992	LC496893	LC496878	LC496908	–	–	Nonaka et al. 2013
*Simplicillium obclavatum*	CBS 311.74	AJ292394	–	AF339517	EF468798	–	–	Nonaka et al. 2013
*Simplicillium spumae*	JCM 39054	LC496871	–	LC496887	LC496917	–	–	Kondo et al. 2020
*Simplicillium spumae*	JCM 39050	LC496869	LC496898	LC496883	LC496913	–	–	Kondo et al. 2020
*Simplicillium subtropicum*	JCM 18180	AB603990	–	LC496880	LC496910	–	–	Nonaka et al. 2013
*Simplicillium subtropicum*	JCM 18181	AB603995	–	LC496881	LC496911	–	–	Nonaka et al. 2013
*Simplicillium sympodiophorum*	JCM 18184	AB604003	–	LC496882	LC496912	–	–	Nonaka et al. 2013

### Taxonomy

#### Ophiocordyceps ramosa

Taxon classificationFungiOphiocordycipitaceae

X. Zou, J. Shen & H.L. Tian sp. nov.

58719685-A061-5162-92F0-FA800E1637CC

860315

Fig. 3

##### Etymology.

“ramosa” in reference to the numerous branches of stromata.

##### Type.

China • Liaoning Province, Tieling City, Xifeng County, Gaojiadian Town (42°42.41'N, 124°26.08'E, 228 m), The material was isolated from Noctuidae (Lepidoptera) larvae, 18 July 2023, Xiao Zou, Huiling Tian, Juan Shen (holotype: GZUIFR GJD03-1; ex-type living culture: GZUIFR GJD03-2).

##### Description.

**Sexual moprh**: Not observed.

**Asexual morph**. Stromata arising from Noctuidae larva buried in soil, multi-branched or partially forked, lignified, light brown, 2.12–3.31 cm long. Colonies on PDA growing very slowly, reaching 22–24 mm in diam after 30 days at 20 °C, flat, spreading radially, grayish-white center, translucent margin; Texture hard and thin, adhering closely to the surface of the culture medium. The reverse exhibited yellowish-brown centers grading into pale yellow, translucent margins. Synnemata growing on PDA medium, grayish white. Two types of conidiogenous cells (A and B) occur singly on the hyphae. Type A:conidiogenous cells unbranched, 20.13–28.97 µm long, with a swollen base, 3.10–3.62 µm wide, tapering sharply into a thin neck, 0.65–1.01 µm wide. Type B: conidiogenous cells branched, multiple (two or more) branches, with a swollen base, the neck gradually tapering to form conidia. Through repeated cycles of swelling and branching, the apical regions narrow again and ultimately produce conidia. Conidia borne directly on the tip of phialides, spherical (5.58–6.67 µm) or sub-spherical (6.47–7.93 × 5.29–6.55 µm), with a mucilaginous sheath.

**Figure 3.**
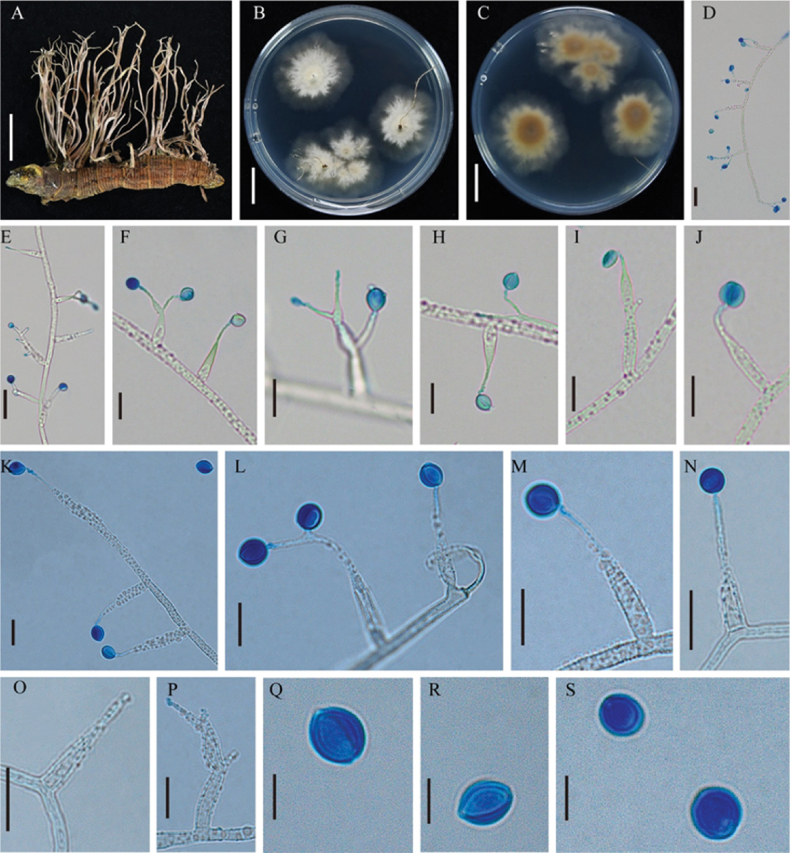
**A**. *Ophiocordyceps ramosa*; **B, C**. Colonial morphology on PDA agar medium for 45 d; **D–N**. Conidiogenous cells and conidia; **O–P**. Conidiogenous cells. Scale bars: 1 cm (**A–C**); 20 µm (**D, E**); 10 µm (**F–P**); 5 µm (**Q–S**).

##### Host.

The larvae of Noctuidae (Lepidoptera).

##### Distribution and Habitat.

China, Liaoning Province, Tieling City. This fungus parasitizes the larvae of Noctuidae (Lepidoptera) within the soil of mixed forests in subtropical humid regions. Its fruiting bodies penetrate the forest litter layer to emerge.

##### Notes.

The conidiogenous cells of *Ophiocordyceps ramosa* were synnematous. Phialides were characterized by a swollen basal part that abruptly narrowed into a slender neck, each producing either single or multiple (two or more) branches. Conidia were spherical or sub-spherical and surrounded by a mucilaginous sheath. Morphologically, *O. ramosa* conformed to the characteristics of the genus *Ophiocordyceps*. Phylogenetic analysis of the genus *Ophiocordyceps* (Fig. 1) indicated that *O. ramosa* formed a separate clade and clustered with *Hirsutella satumaensis* and *H. nodulosa*. However, *O. ramosa* produced two distinct types of conidiogenous cells, which differed significantly in morphology and size from those of *H. satumaensis* and *H. nodulosa* (Table 3). Furthermore, *O. ramosa* parasitizes larvae of Noctuidae, whereas *H. satumaensis* and *H. nodulosa* parasitizes larvae of *Bombyx mori* and mites, respectively, demonstrating distinct differences in host range. Therefore, the morphological features and phylogenetic analyses supported that *O. ramosa* was a new species.

**Table 3.** Morphological comparisons of *Ophiocordyceps ramosa* with closely related species.

Species	Host	Stromata (mm)	Conidiogenous Cells (μm)	Conidia	References
*H. liboensis*	Larvae of Cossidae (Lepidoptera)	Clustered	Base significantly swollen, length 28–30 μm, neck width 1–2 μm, twisting in 2–3 helices at the apex, base width 3–4.5 μm	Fusiform or orange-segmented, in pairs or single, 6–8 × 3–5 μm, with a mucous sheath.	(Zou et al. 2010)
*H. nodulosa*	Lepidoptera, Acari	None	Base swollen, 20–35 × 4 μm, neck often twists in a helix at the apex, neck width 1 μm, with tiny warts	Ellipsoid or shaped like an orange segment, 5–6 × 3 μm, with a mucous sheath	(Minter and Brady 1980)
*H. satumaensis*	Larvae of *Bombyx mori* (Lepidoptera)	Clustered, 3.0–6.5 × 0.5 mm	With Conoid to cylindrical base, size 5–17 × 3–4.5 μm, neck length 7 μm, twisting in a helix at the apex; base with warts	Fusiform or orange-segmented, 5–7.5 × 3–5 μm, with a mucous sheath	(Aoki et al. 1957; Quandt et al. 2014)
*H. subulata*	Larvae of Lepidoptera	Solitary, 15–50 × 0.1–0.3 mm	Phialidic, neck 6–12 µm long, base size (4–8 × 3–5 µm)	Narrowly ellipsoid, in pairs or single, 4–8 × 1.5–2.5 μm, with mucous sheath.	(Hodge 1998)
*Ophiocordyceps campes*	larvae of Lepidoptera	1–3 arising from the host, cylindrical, 50–100 × 1–2, with steriletip	Mono- or polyphialidic. Cylindrical with tiny warts, 21–25 × 3 μm at the base, tapering to a 1 μm wide neck with helical twists at the apex	Hyaline, 1-celled, smooth-walled, lemon-shaped to falcate, arising singly from the apex of the neck, 5–6 × 3–4 μm, embedded in a mucous sheath	(Tasanathai et al. 2020)
*O. longistromata*	larva of Lepidoptera	Several, cylindrical, 50–100×1–2 mm, with sterile tip	Mono- or polyphialidic, hyaline and cylindrical with tiny warts, 22–27 × 2–3 μm at the base gradually tapering to a thin neck, 1 μm wide with twists in a helix at the apex.	Hyaline, smooth, fusiform, arising from the apex of the neck singly, 8–10 × 2–3 μm.	(Tasanathai et al. 2020)
*O. phuwiangensis*	larvae of Lepidoptera	Several, cylindrical, 50–100 × 1–2, with sterile tip	Swollen at the base or slightly flask-shaped, tiny warts, 30–37 × 3 μm, helical twisting at the apex of phialide neck	Hyaline, smooth, ellipsoid to lemon–shaped, arising singly from the apex of the neck, 6–7 × 3–4 μm, surrounded by a mucous sheath	(Tasanathai et al. 2020)
*O. pingbianensis*	Larvae of tiger beetle (Coleoptera)	Geminate, 17–21 × 0.16–0.21 mm	Base obviously swollen, 20.4–31.6 × 3.2–5.2 μm, neck width 1–1.5 μm, twisty and warty at the apex	Solitary, fusiform or oval, 5.3–7.5 × 3.1–4.6 μm, with a mucous sheath	(Chen et al. 2021)
*O. pseudoacicularis*	larvae of Lepidoptera	Solitary to several, cylindrical, 45–70 × 0.5 mm	Monophialidic or polyphialidic, hyaline, smooth. Phialides 12–15 μm, phialide base 6.5–9 × 4–5 μm, phialide neck 4–7 × 0.5–1 μm.	Hyaline, smooth, fusiform, 5–6 × 2 μm, surrounded by mucous sheath.	(Luangsa-ard et al. 2018)
*O. ramosa*	Larvae of Noctuidae (Lepidoptera)	multi-branched, woody, light brown, and partially forked, 2.12–3.31 cm	Unbranched, with a swollen base (3.10–3.62 µm wide) and a tapering neck (0.65–1.01 µm wide), or branch, enlarged at the base, then branch and taper at the neck to produced conidiophores	Spherical (5.58–6.67 µm) or sub–spherical (6.47–7.93 × 5.29–6.55 µm), with a mucilaginous sheath	This study

#### Leptobacillium hepiali

Taxon classificationFungiCordycipitaceae

X. Zou, J. Shen & G.X. Fei sp. nov.

602282AF-3841-5FF8-9582-C51DCD8E3415

860614

Fig. 4

##### Etymology.

“hepiali” refers to its host genus, *Hepialus*.

##### Type.

China • Guizhou Province, Zunyi City, Daozhen County, Guizhou Dashahe Nature Reserve, Yangxi Town (29°04.98'N, 107°34.89'E, 1073 m), The material was isolated from *Ophiocordyceps* spp. of sclerotium (the larvae of *Hepialus* sp.), 28 September 2024, Xiao Zou, Juan Shen & Gangxiang Fei, isolated culture on PDA, culture ex-holotype GZUIFR YX01-2. All isolated strains were deposited at the Institute of Fungal Resources, Guizhou University (GZUIFR), China.

##### Description.

**Sexual morph**. Not found.

**Asexual morph**. Colonies on PDA rather rapid growing, reaching 32–38 mm in diameter in 15 days at 25 °C, circular, raised center, thin hyphae at the edges, aerial hyphae appearing as white downy hairs, reverse yellow. Colonies diameter reached 65–71 mm at 25 °C for 30 days, with a raised white center and pale-yellow coloration from center to margin, reverse yellow in the middle, gradually fading to pale yellow toward the periphery. Conidiogenous cells bearing mainly long solitary phialides. Phialides mainly solitary, columnar, tapering from base to apex, measuring 15.77–47.9 μm in length, with a basal width of 1.38–2.77 μm and apical width of 0.42–0.83 μm. Conidia mostly forming long and slender chains, fusiform, 3.37–5.63 × 0.39–1.60 μm.

**Figure 4.**
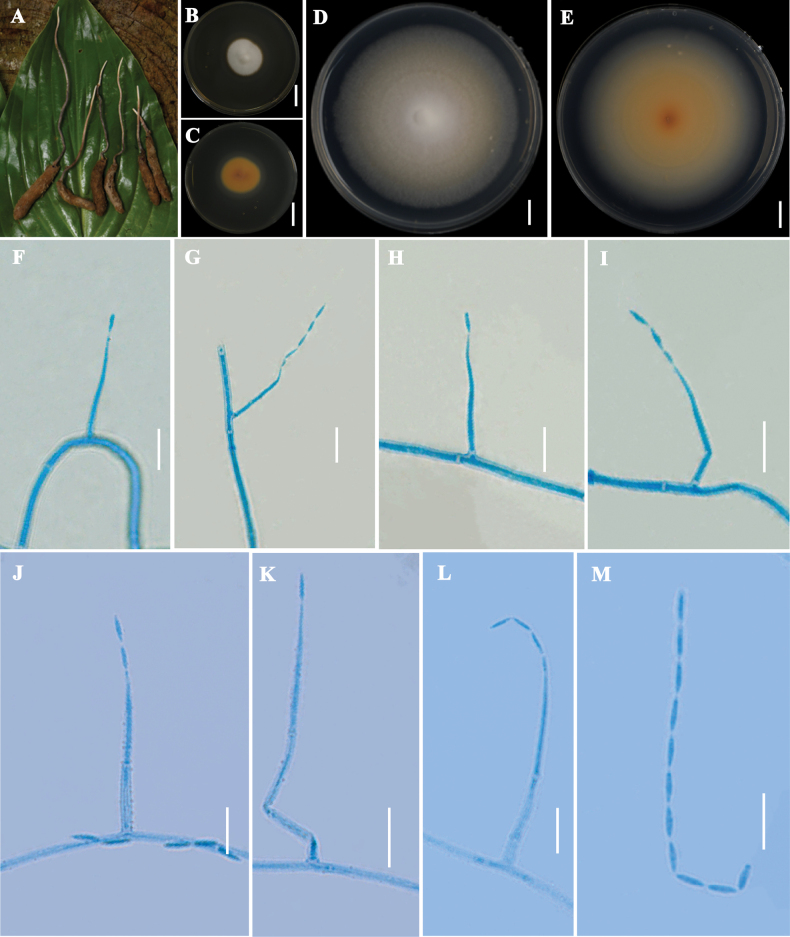
*Leptobacillium hepiali*: **A**. Substrate; **B, C**. Colonial morphology on PDA agar medium for 15 d; **D, E**. Colonial morphology on PDA agar medium for 30 d; **F–L**. Conidiogenous cells and conidia; **M**. Conidiogenous cells. Scale bars: 2 cm (**B, C**); 1 cm (**D, E**);10 µm (**F–M**).

##### Distribution.

China, Guizhou Province: Zunyi City.

##### Substrate.

Sclerotium of *Ophiocordyceps* spp. (within the larvae of *Hepialus* sp.)

##### Additional strain examined.

China • Guizhou Province, Zunyi City, isolated from the larva of *Hepialus* sp., on which the stroma of an *Ophiocordyceps* species had developed, 28 September 2024, Xiao Zou, Juan Shen & Gangxiang Fei, isolated culture on PDA, culture ex-holotype GZUIFR YX01-2. Isolates were deposited at the Institute of Fungal Resources, Guizhou University (GZUIFR), China.

##### Notes.

The genus *Leptobacillium* was proposed by Zare & Gams in 2016 and belongs to the family Cordycipitaceae, its conidiogenous cells were often solitary on the hyphae, producing conidia in chains that were rod-shaped or fusiform. The morphology of *L. hepiali* conformed to the characteristics of the *Leptobacillium* genus. Phylogenetic analysis (Fig. 2) indicated that the new species belongs to the genus *Leptobacillium* and was clustered with *L. latisporum*. The phialides of the new species were solitary, whereas those of *L. latisporum* were branched with two or three (Table 4). Therefore, morphological and phylogenetic analyses supported *L. hepiali* as a new species.

**Table 4.** Morphological comparisons of *Leptobacillium hepiali* with closely related species.

Species	Source, Country	Colony morphology	Phialides (μm)	Conidia (μm)	References
*Leptobacillium cavernicola*	Cave Airborne, Rock art cave airborne organism and Cave Surface sampling, France	White, reverse usually dark brown	Mainly solitary, slender, tapering toward tip, 5.1–27.2 × 1.2–1.7 µm	Forming long, slender chains, narrowly cylindrical to slightly fusiform, some were slightly lemon-shaped, first-formed conidium were usually shorter, obovoid to pyriform with a rounded distal end, 3.1–6.9 × 0.9–1.5 µm	(Leplat et al. 2022)
*L. coffeanum*	Coffee branches, Brazil	White, reverse cream-colored. 30–33 mm (10d)	Mainly solitary, rarely in whorls of 2–3, gradually tapering towards the apex. 11–44 (–70) × 1.0–2.4 μm	Ellipsoidal to fusiform, 2.2–3.8 × 0.8–1.5 μm	(Gomes et al. 2018)
*L. chinense*	Freshwater, China	White, reverse cream to light yellow	Solitary, (6.0–) 15–30(–68.0) × 1.5 µm	Ellipsoidal or oval or cylindrical, 3.5–5.0 × 1.0–1.5 µm, the conidia aggregate into chains, with the apex conidia subspherical or obovoid, 1.5–2.5 × 1.5–2.0 µm	(Liu and Cai 2012)
*L. filiforme*	*Citrullus lanatus* (Cucurbitaceae), Brazil	White, reverse white to yellowish cream. Up to 44 mm (10d)	Solitary, elongate, slightly tapering towards the apex. 9–18 × 1 μm	Fusoid to filiform. 7.2–12.5 × 1 µm	(Crous et al. 2018)
*L. hepiali*.	larvae of Hepialidae (Lepidoptera)	White to Light yellow, reverse yellow to Light yellow, 65–70 mm	Solitary, 15.77–47.9 μm, base width 1.38–2.77 μm, top width 0.42–0.83 µm	Forming long and slender chains, fusiform, 3.37–5.63 × 0.39–1.60 μm	This study
*L. latisporum*	Karst cave Soil, Thailand.	White, reverse greyish orange to orange, white	13.2–40.8 × 2.9–4.8 μm	Shuttle shaped to narrow cylindrical, with single cells forming long chains, 3.9–6.3 × 1.9–3.9 μm	(Preedanon et al. 2023)
*L. leptobactrum* var. *calidius*	*Hemileia vastatrix* on Coffea, Brazil. Living lepidopterous larva, Ghana, Atewa. *Colocasia esculenta*, India.	White to cream, reverse Light yellow to brown.	Solitary, few 1–2 whorls, 18.4–60.0 × 0.7–2.0 µm.	Narrow cylindrical (rod-shaped) to slightly fusiform, 3.0–5.7 × 0.7–1.7 µm.	(Zare and Gams 2016)
*L. leptobactrum* var. *leptobactrum*	*Phlebia tremellosa*, Netherlands, Utrecht. Soil under *Beta vulgaris*, Netherlands, near Groningen. Decaying wood, Poland, Augustów. *Lactarius rufus*, France. Aquarium foam, Japan.	White to cream, reverse Light yellow to yellowish brown	15.8–31.7 × 0.7–1.5 µm Solitary, few 2–3 whorls, 15.8–31.7 × 0.7–1.5 µm	Narrow rod-shaped or narrow cylindrical (rod-shaped), 3.0–6.1 × 0.8–2.1 µm	(Zare and Gams 2016)
*L. longiphialidum*	Spider, China.	White, reverse brown to light yellow	Solitary, 24.01–205.77 × 1.00–2.24 µm	Narrow columnar or spindle shaped, 2.88–4.54 × 1.18–1.95 µm, single celled in chains, with apical conidia elliptical or nearly spherical in shape	(Lu et al. 2025)
*L. muralicola*	White biofilm of mural, China	White, grayish white to greenish white, reverse light yellow, milky white to dark yellow, orange to orange-brown, ochraceous	Solitary, few 1–2 branches, 20.0–45.0 µm long, Base width 1.0–2.0 µm, top width 0.5–0.7 µm	Narrow cylindrical (rod-shaped) to slightly fusiform, 4.5–6.0 × 1.0–2.0 µm	(Sun et al. 2019)
*L. symbioticum*	Prostigmata, sori of *Phakopsora pachyrhizi*, Japan	White, floccose, reverse orange-yellow to orange-brown. 33–53 mm	Mainly solitary, rarely branched with two or three branches, slender, tapering toward tip 7.1–30.6 × 1.6–3.5 μm	Fusiform to cylindrical. 4.0–6.9 × 0.7–1.6 μm	(Okane et al. 2020)
*L. xianyushanense*	*Camellia oleifera* rhizosphere, China	White, irregular floccose surface with divergent cracks, reverse orange to orange-brown, ochraceous, pale luteous, 20–36 mm	Mainly long solitary phialides, rarely with branches of two phialides, tapering toward tip. 24.7–28.5×1.5–1.9 μm	Narrowly cylindrical (rod-shaped) to slightly fusiform. 3.8–5.6×0.5–1.2 μm	(Chang et al. 2024)

## Discussion

Species of the genus *Ophiocordyceps* are taxonomically diverse, morphologically variable, and parasitize a wide range of hosts, with many taxa possessing significant medicinal and ecological value (Lee et al. 2015; De Bekker 2019; Qin et al. 2020; Lysakowska et al. 2023; Wang et al. 2024). Although the fruiting bodies of *Ophiocordyceps* spp. are each formed by a single fungal species, multiple fungal taxa may coexist in natural *Ophiocordyceps* spp. specimens and their microenvironments. High-throughput sequencing has revealed that *Ophiocordyceps sinensis* harbors a rich fungal community (Xia et al. 2015, 2016). Similarly, “multifungal coexistence” has been observed in the sclerotia of *O. indica* and *O. zhenxingensis* (Sharma et al. 2023; Tian et al. 2025). *Paecilomyces hepiali* has been found to coexist with *Ophiocordyceps sinensis* in natural specimens (Zhu et al. 2007). Co-infecting *Hepialus* larvae with two isolated strains of *P. hepiali* and *O. sinensis* conidia significantly enhanced infection efficiency, suggesting that synergistic fungal interaction may be a key mechanism in their occurrence (Li et al. 2016b). Although the mycobiota of *O. sinensis* has been extensively isolated and exploited, research pertaining to other *Ophiocordyceps* spp. remains scarce.

The family Ophiocordycipitaceae currently comprises eight genera, namely *Drechmeria*, *Hantamomyces*, *Harposporium*, *Ophiocordyceps*, *Paraisaria*, *Purpureocillium*, *Tolypocladium*, and *Torrubiellomyces*. *Ophiocordyceps* is the most species-rich genus in Ophiocordycipitaceae and is divided into four major clades (the clade of Hirsutella, the clade of *O. ravenelii*, the clade of *O. sobolifera* and the clade of *O. sphecocephala*). Among them, the *Hirsutella* clade contains six subclades: *H. citriformis*, *H. guyana*, *H. nodulosa*, *H. sinensis*, *H. thompsonii*, and the *Hirsutella* ant pathogen subclade (Sanjuan et al. 2015; Simmons et al. 2015; Qu et al. 2018; Wang et al. 2018). *Ophiocordyceps ramosa* collected in this study exhibits lignified, light brown fruiting bodies consistent with the characteristic tough, fibrous, and often darkly pigmented stromata typical of the genus. The asexual morph of *O. ramosa* is synnematous. The phialides are characterized by a swollen basal part that abruptly narrowed into a slender neck. The conidia are spherical or sub-spherical and surrounded by a mucilaginous sheath, consistent with the morphological characteristics of conidiogenous cells and conidia in *Ophiocordyceps*.

Phylogenetic analysis further showed that *O. ramosa* formed an independent branch (ML bootstrap = 85%, BI posterior probabilities = 0.62) and clustered together with *H. satumaensis* and *H. nodulosa*. *O. ramosa* and *H. nodulosa* possess similar conidiogenous cells with dichotomous branching patterns and a swollen basal part but can be clearly distinguished by their conidia. Specifically, the conidia of *H. nodulosa* are ellipsoidal or shaped like an orange segment, measuring 5–6 × 3 µm (Minter and Brady 1980). In contrast, the conidia of *O. ramosa* are spherical (5.58–6.67 µm) or sup-spherical (6.47–7.93 × 5.29–6.55 µm). Furthermore, the host of *H. nodulosa* is mites (Minter and Brady 1980), while the host of *O. ramosa* is larvae of the family Noctuidae (Lepidoptera). *O. ramosa* and *H. satumaensis* cluster most closely together in the phylogenetic tree. The conidiogenous cells of *H. satumaensis* are similar to the type A conidiogenous cells of *O. ramosa*, as both are unbranched with swollen bases; however, they differ in the morphology of their conidiogenous cells and conidia. In contrast, the conidiogenous cells of *H. satumaensis* are markedly different from the type B conidiogenous cells of *O. ramosa*. Specifically, the conidiogenous cells of *H. satumaensis* measure 5–17 × 3–4.5 μm, and its conidia are fusiform or citrus segment-like, measuring 5–7.5 × 3–5 μm (Aoki et al. 1957; Quandt et al. 2014). The type A conidiogenous cells of *O. ramosa* are 20.13–28.97 × 3.10–3.62 μm, with conidia that are spherical (5.58–6.67 μm) or sub–spherical (6.47–7.93 × 5.29–6.55 μm), whereas the type B conidiogenous cells are branched, which is in sharp contrast to the unbranched conidiophores of *H. satumaensis*. Moreover, *H. satumaensis* specifically parasitizes *Bombyx mori* (Aoki et al. 1957; Quandt et al. 2014).

In this study, a new fungal species was isolated from larvae of *Hepialidae* moths infected by *Ophiocordyceps*, and it was designated as *Leptobacillium hepiali*. This represents the first report of a species of *Leptobacillium* discovered in Guizhou, China. Similarly, Sharma et al. (2023) also obtained a species of *Leptobacillium* through culturable isolation from *Ophiocordyceps indica* (Sharma et al. 2023). The conidiogenous cells of *L. hepiali* are usually solitary on the hyphae, with conidia aggregating at the apices to form slender and elongated chains, morphologically consistent with the diagnostic features of the genus *Leptobacillium*. Phylogenetic analyses revealed that *L. hepiali* is closely related to *L. latisporum* (ML/BI = 100/1), yet *L. hepiali* forms a distinct monophyletic lineage (BI posterior probability = 1, ML bootstrap = 100%). Morphologically, the conidia of *L. hepiali* occur singly, with phialides being unbranched, in contrast to the typically 2–3 branched phialides of *L. latisporum*. Furthermore, the conidia of *L. hepiali* are comparatively smaller (3.37–5.63 × 0.39–1.60 μm) than those of *L. latisporum* (3.9–6.3 × 1.9–3.9 μm). Therefore, both morphological distinctions and phylogenetic evidence support the recognition of *L. hepiali* as a novel species.

Through analyses of morphological features and phylogenetic relationships, this study identified a novel entomopathogenic fungus, *Ophiocordyceps ramosa*, and simultaneously isolated a new fungal species, *Leptobacillium hepiali*, from insect hosts infected by *Ophiocordyceps*. These findings expand the known species composition of *Ophiocordyceps* and *Leptobacillium*, and provide a new and tangible case for exploring the widespread “multifungal coexistence” phenomenon within *Ophiocordyceps*.

## Supplementary Material

XML Treatment for Ophiocordyceps ramosa

XML Treatment for Leptobacillium hepiali

## Additional information

### Use of AI

The authors utilized ChatGPT for the purpose of translating and polishing the initial manuscript draft from Chinese to English. Following the translation, the authors thoroughly reviewed and edited the content to ensure accuracy and take full responsibility for the published work.

### Author ORCIDs

Juan Shen https://orcid.org/0009-0002-3354-2327

Wei Gou https://orcid.org/0009-0000-3451-3566

Hui-Ling Tian https://orcid.org/0009-0001-0385-9721

Gang-Xiang Fei https://orcid.org/0000-0002-4493-8397

Jing-Qiang Guan https://orcid.org/0000-0002-6312-2545

Chun-Yan Long https://orcid.org/0009-0000-9790-8258

Xiao Zou https://orcid.org/0000-0002-3666-5536

### Data availability

All of the data that support the findings of this study are available in the main text.
